# Acceptability and Adherence to Ready-to-Use Therapeutic Foods (RUTFs) Treatment in Cases of Moderate and Severe Acute Malnutrition in Children from Rural and Indigenous Communities in Mexico

**DOI:** 10.3390/nu18030444

**Published:** 2026-01-29

**Authors:** Edgar Arturo Chávez Muñoz, Ana Lilia Lozada Tequeanes, Selene Pacheco Miranda, Leonel Dorantes Pacheco, Mariana Castañeda Barrios, Alexander Cueva-Chamba, Anabelle Bonvecchio Arenas, Matthias Sachse, Cecilia de Bustos

**Affiliations:** 1 Nutrition and Health Research Center (CINyS), National Institute of Public Health of Mexico (NIPH), Avenida Universidad 655, Santa María Ahuacatitlán, Cuernavaca 62100, Mexico; nutpedia.chavezm@gmail.com (E.A.C.M.); cinys20@insp.mx (A.L.L.T.); selene.86517@gmail.com (S.P.M.); ldorantespa@gmail.com (L.D.P.); mariancstb@gmail.com (M.C.B.); alexander.cueva@insp.edu.mx (A.C.-C.); 2 United Nations International Children’s Emergency Fund (UNICEF) of Mexico, Avenida Paseo de la Reforma 645, Lomas de Chapultepec, Miguel Hidalgo, Ciudad de México 11000, Mexico; msachse@unicef.org (M.S.); cdebustos@unicef.org (C.d.B.)

**Keywords:** acceptability, adherence, moderate and severe acute malnutrition, RUTF, children, Mexico

## Abstract

**Background/Objectives**: Infant acute malnutrition increases the risk of morbidity and mortality but also has adverse effects on growth, cognitive development, and quality of life. Ready-to-use therapeutic foods (RUTFs) represent the standard treatment in moderate (MAM) or severe acute malnutrition (SAM) cases, but acceptability and adherence in culturally diverse settings remain poorly understood. To evaluate the acceptability and adherence to RUTF treatment among children with MAM or SAM in rural and indigenous communities of three Mexican states. **Methods**: We conducted a mixed-methods study in Chihuahua, Guerrero, and Chiapas between February and August 2024. A total of 66 children aged 6–59 months with confirmed MAM or SAM diagnoses were enrolled, with 48 completing the study. Acceptability was assessed by weighing leftovers of the consumption RUTF sachet and using 5-point hedonic scales evaluating taste, texture, appearance, and smell. Adherence was measured by comparing consumed versus prescribed RUTF sachets. Semi-structured interviews with primary caregivers explored perceptions, barriers, and facilitators of RUTF use. **Results**: Consumption-based acceptability was achieved by 85% of participants. On the 5-point hedonic scale, 77% of participants rated RUTF as pleasant or very pleasant. Overall treatment adherence was adequate (≥70%) in 79.2% of cases. Qualitative information revealed primary caregivers’ perceptions about weight gain and increased appetite as benefits. **Conclusions**: Since acceptability and adherence were adequate and a high score was obtained on the hedonic scale, which led to an improvement in the nutritional status of MAM or SAM cases, the feasibility and acceptability of RUTFs in highly vulnerable community contexts in Mexico was reaffirmed.

## 1. Introduction

Infant acute malnutrition (IAM) represents a critical global health challenge, affecting approximately 45 million children under five years of age [1]. IAM is classified as moderate acute malnutrition (MAM) when weight-for-height/length z-scores (WHZ/WLZ) fall between −3 and −2 standard deviations (SD) and as severe acute malnutrition (SAM) when z-scores are located <−3 [2]. This condition not only increases the risk of morbidity and mortality but also has short and long-lasting adverse effects on growth, cognitive development, and quality of life [3].

In Mexico, data from the 2023 National Health and Nutrition Survey (ENSANUT) indicate that 124,201 children under five years of age (1.2%) are affected by acute malnutrition [4]. This prevalence is notably higher among children living in rural areas and households where an indigenous language is spoken, reaching double the prevalence (2.3%) [4]. States such as Guerrero, Chiapas, and Chihuahua, located in southeastern and northern Mexico, concentrate a high proportion of indigenous populations and present some of the highest IAM rates in the country [5]. These disparities reflect persistent structural inequalities, including limited access to health services, safe water, and food security [6].

For addressing IAM in resource-limited settings, ready-to-use therapeutic foods (RUTFs) are internationally recommended as the standard treatment for children aged 6 to 59 months with uncomplicated MAM or SAM [2,7,8]. The RUTFs are prioritized in outpatient schemes due to high energy density (500 kcal/92 g sachet), complete profile of essential micronutrients, as well as adequate amounts of macronutrients, ease of use requiring no preparation, and long shelf life, making them ideal for resource-limited settings [9].

The World Health Organization (WHO) supports differentiated dosing protocols, with one sachet/day (500 kcal) for MAM and two sachets/day (1000 kcal), optimizing both nutritional recovery and resource utilization in large-scale programs [2,10,11].

Although the efficacy of RUTF has been widely documented in international studies [9,10,11], there is a critical lack of evidence from Latin America, where food cultures, caregiving practices, and perceptions of peanut-based therapeutic products differ substantially. In Mexico, RUTF is not included in the National Catalogue of Medicines, and its use depends on external procurement, limiting its integration into routine child health services. Rural and indigenous communities face unique structural barriers, including food insecurity, limited access to safe water and health services, linguistic diversity, and different complementary feeding practices [6], which are expected to influence adherence patterns, caregiver decision-making, and the acceptability of RUTFs in ways that differ from previously studied contexts [8].

Acceptability refers to the extent to which an intervention is perceived as appropriate by recipients and providers [12], can be influenced by sensory factors (appearance, taste, texture, smell, and palatability), social aspects (eating habits), and operational delivery mechanisms. For its part, adherence is defined as compliance with RUTF treatment consumption in relation to the prescription sachets established by health personnel for the entire duration of treatment and may be affected by family dynamics, logistical challenges, and health system factors [13]. Generating empirical evidence on these dimensions, we studied a real-world experience of RUTF use in highly vulnerable Mexican populations and provided actionable information to support future budget allocation, inclusion of RUTF in national clinical guidelines, and culturally adapted implementation strategies aimed at reducing IAM in Mexico.

Therefore, this study aimed to evaluate the acceptability and adherence to treatment with RUTF in children aged 6 to 59 months who were diagnosed with MAM or SAM in rural and indigenous communities of three Mexican states.

## 2. Materials and Methods

### 2.1. Study Design and Location

A mixed-methods study with explanatory sequential design [14] and complementarity [15] was conducted to evaluate the acceptability and adherence to treatment with RUTFs among children in rural and indigenous communities. The study was conducted across three Mexican states characterized by high indigenous populations and high levels of marginalization: Chihuahua, Guerrero, and Chiapas [4]. Each child was followed through a minimum of three study contacts (baseline, mid-treatment, and end of treatment), with additional visits scheduled when required until completion of the RUTF protocol.

### 2.2. Subjects and Recruitment

Eligible participants were children aged 6–59 months diagnosed with MAM or SAM, with a positive appetite test and no medical complications requiring inpatient care [16]. Inclusion criteria also required full-term birth (≥37 weeks) and previous exposure to peanuts and complementary feeding. The mother or primary caregiver had to be of legal age (≥18 years old) and speak Spanish (>80%). On the other hand, the main exclusion criteria were being beneficiaries of another program that provided nutritional supplements and/or having physical or mental limitations to answer the questionnaires/interviews or take anthropometric measurements.

**Figure 1:**
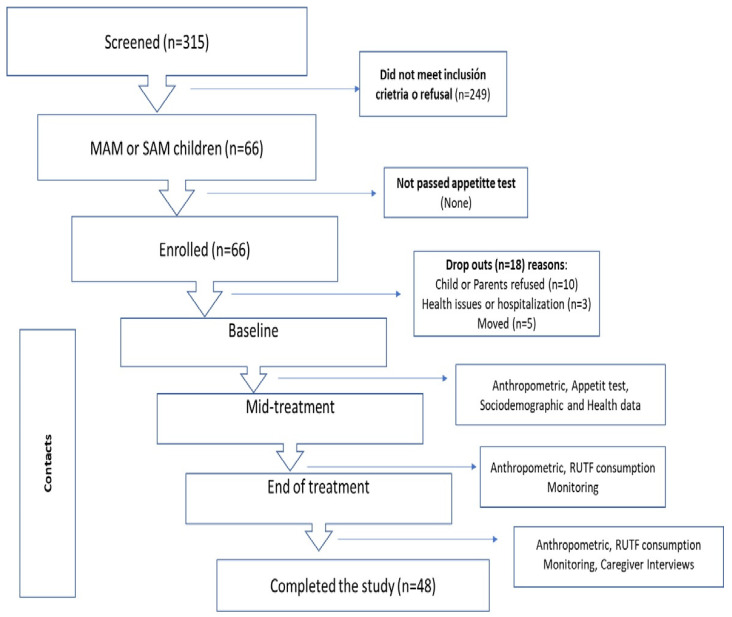
Study flowchart.

Potential participants were recruited at the primary healthcare unit, where the research team had previously trained healthcare staff in the identification of IAM and in the use of RUTF treatment. The healthcare staff pre-screened children for MAM or SAM using Mid-Upper Arm Circumference (MUAC) [17] to allow better detection of children with low WHZ/WLZ. This approach generated a short list of children at high risk of MAM or SAM. The screening process consisted of confirming MAM or SAM based on anthropometry (WHZ/WLZ), physical examination (nutritional oedema), and appetite test. All mothers or primary caregivers of children meeting the inclusion criteria were invited to participate in the intervention study, and parents who gave informed consent were finally enrolled in the study (Figure 1).

A convenience sample of approximately 20 children per state was targeted to provide an exploratory overview of acceptability and adherence in highly vulnerable and hard-to-reach populations, acknowledging the operational challenges of identifying and retaining children with acute malnutrition in these settings [18].

Given the vulnerable conditions of the study population and the high level of participation by the mother or primary caregiver in the study, cash incentives ($10 USD) were provided for each follow-up of the child in the study until the end of treatment.

### 2.3. Ethical Considerations

This study was approved by the Ethics, Research and Biosafety Committees of the National Institute of Public Health (INSP) of Mexico with reference number CI. 1753. Written informed consent was obtained from all participating mothers or primary caregivers prior to enrollment.

### 2.4. RUTF Intervention

The intervention consisted of the provision of RUTF according to nutritional status at admission, individualized counselling on adequate RUTF administration and complementary feeding practices, and follow-up and monitoring by trained primary healthcare personnel every 15 days. At enrollment, caregivers received a basic support kit to facilitate adherence to the intervention.

According to WHO recommendations [2], treatment with RUTF was assigned according to the severity of acute malnutrition: MAM cases received 7–15 sachets (1 per day), covering 1 or 2 weeks of treatment, while SAM cases received 15–30 sachets (2 per day). The estimated treatment duration ranged from 30 to 60 days and was adjusted by healthcare staff based on individual clinical progress and contextual factors.

RUTF was distributed by healthcare staff at each primary health care unit during every follow-up visit, scheduled approximately every 15 days, and the supply was provided in a recyclable cloth bag.

During these visits, caregivers also received counselling and printed educational material (Supplementary File S1) covering instructions for daily intake, management of potential side effects, recommendations in case of adverse events, avoidance of sharing RUTFs with other household members, consumption of other foods only after the daily RUTF dose, and continuation of breastfeeding.

### 2.5. Acceptability and Adherence Measurements

#### 2.5.1. Consumption-Based Acceptability

To estimate the percentage of RUTF intake, the sachets were numbered, weighed, and recorded before and after consumption using standardized forms and following biosafety measures. Mothers and primary caregivers were asked to return all used and unused sachets at each follow-up visit, and any remaining content was weighed to calculate actual intake. Consumption was considered adequate when the mean intake was ≥50% of the provided sachets, based on previous studies using similar nutritional supplements [19,20,21].

#### 2.5.2. Hedonic Scale Acceptability

A 5-point hedonic (or smiley face) scale was used to evaluate subjective perception by children and their mothers/primary caregivers about organoleptic characteristics of the RUTF, such as appearance, smell, taste, texture, and overall likability. The research team applied this scale to mothers/primary caregivers during follow-up visits using a printed format with faces illustrating the different response options. Responses were interpreted from “very unpleasant” (1 point), “unpleasant” (2 points), “neutral” (3 points), and “pleasant” (4 points) to “very pleasant” (5 points). High acceptability was defined as scores of 4 or 5 across all assessed attributes in each follow-up visit [22].

#### 2.5.3. Adherence to Treatment

Adherence was calculated by comparing the number of RUTF sachets consumed with the prescribed treatment for MAM or SAM. At each visit, caregivers returned all sachets, which were counted and verified against prescribed doses recorded by healthcare staff. Consumption diaries were reviewed to complement this data (see Section 2.6.4). Adequate adherence was defined as consumption between 50% and 90% of prescribed doses throughout the treatment period [23].

### 2.6. Procedures and Data Collection

Prior to data collection, research teams were extensively trained on interview and survey skills, the content of each questionnaire, and the use of the RedCap platform (Research Electronic Data Capture Software 14.8.3) [24]. They were also retrained and standardized to conduct child anthropometric measurements and to weigh the leftovers of RUTF sachets and record them.

Data collection was conducted according to a standardized sequence of procedures: (1) anthropometric measurements, (2) appetite test, (3) sociodemographic and health data collection, (4) RUTF consumption monitoring, and (5) mother/primary caregiver interviews. Each of these components is described in detail below (see Figure 1).

#### 2.6.1. Anthropometric Assessment

At recruitment, height or recumbent length was measured to the nearest 0.1 cm, and weight was measured to the nearest 0.1 kg using standardized techniques. MUAC was measured to the nearest 1 mm using standardized measuring tapes [25,26]. All measurements were performed in duplicate by trained nutrition professionals and were repeated at midterm and at the end of the study. All children were screened for oedema using standardized techniques [18].

The average of duplicate measurements was used for all analyses. Although small variations in weight close to anthropometric cutoffs could theoretically influence nutritional classification, the use of duplicate measurements, standardized procedures, and trained personnel minimized random measurement error. In addition, nutritional status was determined using multiple criteria (weight-for-height, MUAC, and bilateral pitting oedema), thereby reducing the likelihood of misclassification due to minor imprecision in any single indicator.

#### 2.6.2. Appetite Test

The appetite test was conducted according to WHO guidelines for the management of IAM [25]. Caregivers were asked to offer one RUTF sachet to the child, while the healthcare staff observed consumption for up to 20–30 min. Duration of intake, amount consumed, the observed reaction of the child, and information on the child’s normal appetite were recorded. An appetite test was considered a failure if the child refused the RUTF or consumed less than 50% of the sachet and was judged to have a very poor appetite [27].

At each contact, healthcare staff also verified the absence of nutritional oedema and medical complications.

#### 2.6.3. Sociodemographic and Health Data

At baseline, a questionnaire was administered to collect data on household sociodemographic status, as well as information covering the previous 3 months of children’s feeding practices, use of micronutrient supplements, morbidity history, and immunization status. Data were recorded on tablets using RedCap software [24]. At the end of follow-up, anthropometric measurements, consumption diaries, and remaining RUTF sachets were collected.

#### 2.6.4. RUTF Consumption Monitoring

Mothers or primary caregivers were provided with consumer diary sheets—and an explanation of their use—to record the frequency and timing of RUTF consumption each day. If the mother/primary caregiver did not know how to write, a member within the family was identified who could participate with this very simple recording activity. A field worker at each follow-up at the health unit collected these recordings, as well as information on intra-household sharing of the RUTF, child morbidity, or side effects presented. Also, the field worker counted the remaining sachets and weighed any open sachets to calculate the total amount of RUTF product consumed.

Mothers/caregivers were provided with basic materials, including labels, containers, and storage bags, to preserve leftover RUTFs until collection. When it was possible, health personnel and field workers maintained regular communication with the mothers/primary caregivers through texting or phone calls for reminders for the indications and/or for the follow-up appointments. Additionally, as mentioned before, during these follow-up contacts, the research team administered the printed format of hedonic scale to assess mothers/primary caregivers’ and children’s perception of the RUTF’s organoleptic characteristics.

#### 2.6.5. Mothers and Primary Caregivers Interviews

At the final contact, semi-structured interviews were conducted with mothers or primary caregivers of children to explore perceptions, barriers, and facilitators related to RUTF use. The interview guide was developed based on the Theoretical Framework of Acceptability (TFA) by Sekhon and colleagues [28], which included product experience, perceived health and nutritional changes, implementation barriers, and understanding of usage instructions.

### 2.7. Statistical Analysis

Quantitative information was entered into a digital questionnaire in real time, extracted into datasets, and cleaned. General characteristics of participants, including household and maternal information, child morbidity, among others, were examined using descriptive statistics; means (±SD) were calculated for continuous variables and percentages for categorical variables.

Maternal sociodemographic variables were categorized as follows: women’s age (years); marital status (single, married, and cohabiting); educational level (basic, middle/high school, and university or higher); and occupation (employed, housewife, farmer, merchant, or other). Indigenous speakers say yes or no.

Socioeconomic status (SES) [29] was classified by estimating the level of satisfaction of the most important needs of the household, considering six characteristics and household assets. SES was classified according to the Mexican Association of Market Research Agencies (AMAI in Spanish). The six variables were Human Capital, Practical Infrastructure, Connectivity and Entertainment, Healthcare Infrastructure, Planning and Future, and Basic Infrastructure and Space. Finally, SES was grouped into four categories: high, medium, low, and very low.

Anthropometric measurements were conducted at baseline and subsequent follow-up visits, including weight and Mid-Upper Arm Circumference (MUAC). Z-scores for weight-for-height/length (WHZ/WLZ) and height/length-for-age (HAZ/LAZ) were calculated using the 2006 WHO growth standards [30]. The diagnosis of MAM was based on WHZ < −2 SD or MUAC < 12.5 cm, while SAM was defined as WHZ < −3 SD or bilateral edema +/++. Also, MUAC classification thresholds followed WHO guidelines: <11.5 cm for SAM and 11.5–12.5 for MAM, 11.5–12.5 cm for MAM, and 12.5–13.5 cm for mild acute malnutrition [2,21].

Age and anthropometric measurements were treated as continuous variables, while sex was analyzed as a categorical variable (boys and girls). A dichotomous variable was created for the presence or absence of MAM or SAM. Comparisons of continuous variables were performed using analysis of variance (ANOVA), whereas categorical variables were compared using Pearson’s chi-square test or Fisher’s exact test when appropriate. All statistical analyses were conducted using Stata version 14.5, and a *p*-value < 0.05 was considered statistically significant.

### 2.8. Qualitative Information

Qualitative information was transcribed verbatim, coded, and thematically analyzed [31] using Atlas.ti version 24. The qualitative analysis reached theoretical saturation, as the information obtained in the interviews did not contribute any new categories or dimensions relevant to the analytical framework. At that point, the findings became recurrent and consistent, indicating that the categories identified were sufficiently developed and that additional data did not offer substantial variations. Therefore, it was considered appropriate to conclude the information-gathering phase. Finally, results from both quantitative and qualitative components were used to identify different facets of the same phenomenon, using the complementarity approach proposed by Green et al. [15].

## 3. Results

### 3.1. Characteristics of the Study Population

A total of 315 children were screened, and 66 met inclusion criteria and had confirmed diagnoses of MAM or SAM. They were distributed as follows: Guerrero n = 31 (47%); n = 22 Chiapas (33%); and Chihuahua n = 13 (20%). Of these, n = 18 (27.2%) were lost to follow-up and/or their parents did not consent or refused to continue in the study (Figure 1).

The highest attrition was observed in Chihuahua (8/13) versus Chiapas (2/22); however, these differences were not statistically significant. No significant differences were found between children who dropped out and those who remained in the study in terms of age or anthropometric measurements (weight, length/height, and MUAC; *p* > 0.05) (Appendix A Table A1). Regarding mothers/primary caregivers, the mean age was 29 (±5.7) years, over 80% (56/6) reported being housewives, and more than half had basic schooling or less. Almost half (29/66) reported speaking an indigenous language. In addition, 66% of the households in the sample studied (43/66) reported a low or very low SES (Table 1).

A total of 48 children completed the RUTF treatment and were included in the analytical sample. Of these, a total of 28 mothers of primary caregivers were interviewed, with a mean age of 29.1 (±5.7) years. Most had completed middle or high school education (15/28), and a few had attained a bachelor’s degree (3/28). More than 80% (23/28) reported being housewives. This subsample represented 42.4% (28/66) of the total recruited participants and were those who had a semi-structured interview about their experiences of using RUTFs.

Among the 66 enrolled children, the mean age was 23.5 (±15.4) months; 63.0% were between 6 and 24 months, and the majority were male (53%, 35/65). Anthropometric measurements showed an average weight of 8.3 (±2.1) kg and an average length of 77.8 (±11.6) cm. Only 0.9% (6/66) had weight/length (WLZ) scores < −3; the overall mean MUAC was 12.2 (±0.5) cm (Table 1).

### 3.2. Consumption-Based and Hedonic Scale Acceptability

**Figure 2:**
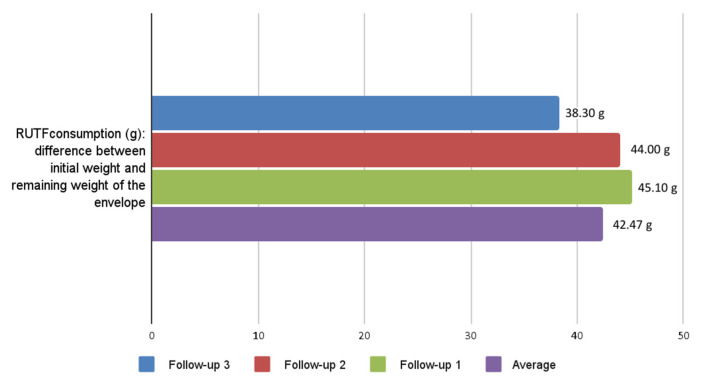
RUTF consumption-based acceptability among children with MAM or SAM.

Consumption-based acceptability, defined as >50% of the prescribed RUTF intake (average leftovers per sachet: 44.0 [±29.5] g), was achieved by 85.4% of participants throughout the study period (Figure 2).

Analysis by state revealed significant differences, with Chiapas and Guerrero achieving consumption rates above 85% of prescribed RUTF, compared to 50% in Chihuahua.

Hedonic scale assessment among the 48 participating children revealed high acceptability across all sensory attributes, with scores of “pleasant” or “very pleasant”: 81% for texture, 86% for taste, 77% for appearance, 84% for smell, and 92% for overall liking. Overall, more than 77% of participants adequately accepted the RUTF. Mothers or primary caregivers rated the RUTF with average scores above 83% for texture, 84% for appearance, 81% for smell, 74% for taste, and 90% for overall liking (Table 2).

### 3.3. Adherence to Treatment

**Figure 3:**
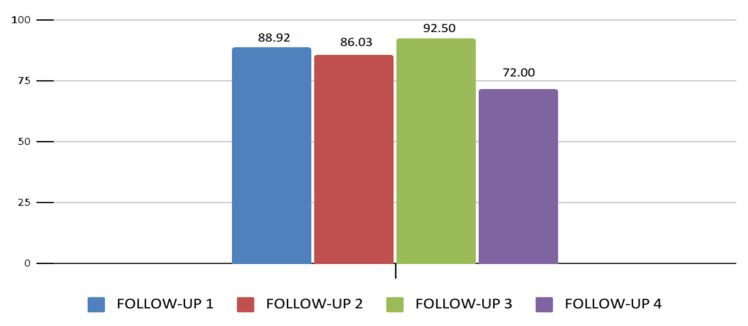
Adherence (Adherence was calculated based on the number of RUTF sachets consumed relative to the number prescribed. Follow-up 1: n = 51; Follow-up 2: n = 46; Follow-up 3: n = 20; Follow-up 4: n = 5 (*p* > 0.05).) to RUTF treatment in children with MAM and SAM.

Adherence to the prescribed RUTF treatment, defined as ≥70% consumption, was achieved throughout the follow-ups by participants who completed treatment. Adherence decreased from 88.9% adherence to treatment at the first follow-up to 72% at the last follow-up (Figure 3). Adherence rates also varied significantly by state: Guerrero 85.1%, Chiapas 94.6%, and Chihuahua 71.3% during the intervention (Figure 3).

The most frequent reasons for low adherence reported were forgetting to give the child RUTF (5–15%), voluntary treatment interruption, child refusal, and insufficient training of healthcare personnel in anthropometric assessment and the management of acute malnutrition, which hindered appropriate case detection and follow-up (Appendix A Table A2).

### 3.4. Anthropometric Results

Mean body weight increased progressively during follow-up, with an average weight gain of 1.07 kg between baseline and the end of treatment. Mean weight values were as follows: baseline (n = 65) 8.33 kg SD; follow-up 1 (n = 51) 8.81 kg SD; follow-up 2 (n = 45) 9.40 kg SD; follow-up 3 (n = 23) 8.72 kg SD; and follow-up 4 (n = 10) 8.67 kg SD.

Similarly, MUAC increased by an average of 1.04 cm over the 8-week treatment period, with the following values: baseline (n = 63), 12.27 cm; follow-up 2 (n = 46), 12.94 cm; follow-up 3 (n = 23), 13.31 cm; and follow-up 4 (n = 10), 13.27 cm.

Although these trends did not reach statistical significance (*p* > 0.05), child recovery was determined based on MUAC or WHZ measurement and completion of RUTF treatment according to your initial diagnosis (Appendix A Table A1).

### 3.5. Interviews with Mothers or Primary Caregivers

Testimonies revealed that mothers or primary caregivers attempted to administer the RUTF treatment and follow the prescribed dosing protocols (Appendix A Table A2). Families developed adaptive strategies to overcome children’s initial rejection of RUTF, such as mixing it with preferred foods and establishing consistent feeding routines. In addition, mothers or primary caregivers used visual reminders and adapted storage to children’s preferences, which supported treatment adherence.

Perceived treatment benefits included visible weight gain, improved appetite, and increased energy levels among children. Mothers or primary caregivers consistently reported that the taste and texture of RUTF were acceptable. These qualitative findings were consistent across the three states, and many families reported an overall improvement in the child’s health. The treatment was described as easy to administer and well-received by the children, with no need for coercion (Appendix A Table A2).

In addition, the analysis suggested that the visual educational materials provided during the program facilitated caregivers’ understanding of RUTF identification and dosing instructions and helped to address common questions regarding treatment administration, which may have contributed to consistent use of the product throughout the study period.

Caregivers generally reported that the peanut-based flavor of RUTF was well accepted by children and did not generate significant cultural resistance. Interview data suggested that the sweet and creamy taste was perceived as similar to other locally consumed snacks or spreads, facilitating its integration into daily feeding routines. However, some caregivers indicated that the texture and sweetness differed from traditional complementary foods, which occasionally required an adaptation period during the first days of treatment. These findings highlight that, despite cultural differences in dietary habits, the sensory characteristics of RUTF were largely compatible with local food preferences in the studied rural and indigenous communities.

Adverse effects were reported, mainly gastrointestinal symptoms, with diarrhea being the most common. However, mothers or primary caregivers usually attributed these symptoms to environmental factors, such as water quality or concurrent illnesses, rather than RUTF consumption. Isolated cases of vomiting, constipation, or stomach discomfort were also mentioned. These effects were generally transient and did not compromise overall acceptability or adherence. Discontinuation due to adverse effects occurred in only one case of persistent vomiting, reinforcing the good tolerability of RUTF and caregivers’ willingness to continue its use (Appendix A Table A2).

In terms of recovery, all children who completed the study improved from SAM or MAM to being at risk of malnutrition or normal nutritional status, and families expressed willingness to continue RUTF use if accepted by the child. Mothers or primary caregivers also highlighted the importance of maintaining consistent taste and texture of RUTF and suggested combining it with familiar foods to enhance acceptance and treatment effectiveness (Appendix A Table A2).

## 4. Discussion

This study found high levels of acceptability and adequate adherence to RUTF treatment among children aged 6 to 59 months diagnosed with MAM or SAM in rural and indigenous communities in Mexico. Consumption-based acceptability was adequate in more than 85% of children, and over 77% rated the product as “pleasant” or “very pleasant” on the hedonic scale. Treatment adherence (≥70% of prescribed sachets) was achieved by nearly 80% of participants and was reflected in gradual improvements in anthropometric indicators. Qualitative findings supported these results, revealing positive caregiver perceptions of RUTF and identifying practical strategies to overcome initial barriers to consumption.

Our findings demonstrate high RUTF acceptability and adequate adherence, with the majority of participants achieving adequate consumption levels regarding the prescribed intake/treatment. This was corroborated by the measurement of the hedonic scale, revealing favorable ratings across all sensory attributes as “pleasant” or “very pleasant” among the sample studied.

These findings are consistent with previous studies reporting high acceptability of RUTF and its positive impact on nutritional recovery. For example, Thapa et al. (2017) [32] in northern India documented high acceptability and efficacy of a locally produced RUTF (Nutreal), emphasizing that its lack of preparation requirements facilitated its use. Similar to our findings, adverse effects were minimal, with diarrhea reported in only 3 of 112 children.

In contrast, a study conducted in Bangladesh in 2013 [33] evaluating acceptability of Pumply’Nut among children aged 6 to 59 months reported that 60% of caregivers reported difficulties with acceptance related to taste (43%) and consistency (31%), and 64% of caregivers attributed side effects to Pumply’Nut, including nausea, vomiting, diarrhea, bloating, and pain. In contrast, our study showed high consumption-based acceptability and favorable hedonic ratings, along with a lower frequency of adverse effects throughout the intervention.

Our results also differ from previous research conducted in urban communities in central Mexico assessing the acceptability of small-quantity lipid-based nutrient supplements (SQ-LNS), where acceptability levels ranged from 31.5% to 44% among children aged 7 to 24 months in both initial and final phases of the study [34]. That study focused on malnutrition prevention rather than treatment and included children who were not acutely malnourished, whereas our study targeted children with MAM or SAM in rural and indigenous communities characterized by high food insecurity [35].

Analysis by states revealed important cultural barriers, particularly in Chihuahua (in northern Mexico), where limited traditional consumption of peanuts may have contributed to lower acceptability rates and higher dropouts during the study, which has already been documented in other contexts [33]. This last, due to Chihuahua, seems unfamiliar with the sweet taste and texture characteristics because some mother/primary caregivers noted that the flavor of RUTF was unfamiliar to the children, associating it with the presence of peanuts, which are not commonly consumed in their diet. Instead, in Chiapas and Guerrero (southern and south-central Mexico), peanuts were more common in the diet. Mothers and primary caregivers mentioned they usually offered them at older ages, not during complementary feeding [36]. Furthermore, in Chihuahua, especially food insecurity, adverse socioeconomic conditions, geographic dispersion, and family customs limited the regular incorporation of peanut-based RUTF, which was reflected in lower acceptability and biggest dropouts of the study.

At regional and global levels, evidence of adherence to RUTF treatment remains scarce, particularly in Latin America and the Caribbean. A 2019 Cochrane meta-analysis [7] concluded that adherence outcomes are unclear, noting that few studies included adherence as an outcome and also mentioned the possibility that RUTF may be sold or shared with siblings or other family members. In our study, intrafamilial sharing was rarely reported, with only one documented case across all states. This low level of sharing may be explained by the fact that RUTF is registered in Mexico as a therapeutic product rather than a food [37], and health personnel were specifically trained to instruct families that it should be consumed only by children diagnosed with acute malnutrition.

This latter may be because malnutrition remains a problem in these socially and economically vulnerable populations, and treatment with RUTF appears to be a nutritional option for children diagnosed with MAM or SAM [38], hence the importance of counselling to continue breastfeeding (from birth to at least 24 months) and to maintain adequate infant and young child feeding (IYCF) practices after finishing the treatment [13].

Although overall adherence to RUTF treatment was high, caregivers reported specific challenges during the initial weeks of treatment. These included difficulties related to transportation to health facilities, limited availability of safe water for hygiene practices, and concerns about digestive discomfort, such as diarrhea, vomiting, or abdominal bloating, at the beginning of RUTF consumption. Qualitative interviews indicated that these initial symptoms occasionally generated doubts about continuing treatment, particularly among caregivers with limited prior exposure to therapeutic foods. In parallel, health personnel highlighted that poor hygiene conditions and lack of safe water in some communities required attention from local social and sanitation programs. Addressing these barriers through enhanced counselling and culturally adapted communication strategies could further strengthen adherence, while the observed anthropometric improvements support the effectiveness of community-based RUTF treatment in these Mexican participants.

This study has some limitations, such as it was not possible to reach the expected sample size in all three states, because most of the MAM/SAM cases appear suddenly, which is difficult to capture, especially in vulnerable population from remote areas and predominantly speak indigenous languages. Although community members supported translation, linguistic barriers remained, particularly in Chihuahua, limiting effective communication, accurate data collection, and contribution to higher dropout rates among indigenous language–speaking caregivers. In addition, the specific RUTF formulation used (Plumpy’Nut with a peanut–soy blend) may limit results generalizability to other RUTF products or locally produced alternatives. The version currently available in Mexico, supported by UNICEF, contains a blend of peanuts and soy flour, which may alter its flavor; this differs from versions made exclusively or predominantly with peanuts. Future studies should evaluate RUTF formulations made solely from peanuts or, as is already being done in other countries, locally produced RUTF using regionally familiar ingredients [39].

Despite these limitations, this study has several strengths. To our knowledge, this study is the first aimed at evaluating the acceptability and adherence to treatment with RUTF among children aged 6 to 59 months diagnosed with MAM or SAM in rural and indigenous communities across three Mexican states, contributing novel evidence to the limited literature on RUTF implementation in Latin American contexts.

As another strength of this study to the RUTF evidence, it is identified that, first, this represents among the first systematic evaluations of RUTF acceptability and adherence in Mexican populations with MAM or SAM condition. Second, the diverse geographic and cultural contexts of the sample was studied, including northern (Chihuahua) and southern regions (Guerrero and Chiapas), with historically high rates of IAM prevalence. Third, it assessed acceptability and adherence from the perspective of mothers/primary caregivers to know in depth their experiences that can directly influence adherence and acceptability of RUTF as well as the therapeutic response of the children.

## 5. Conclusions

Based on these findings, treatment of IAM using RUFT is feasible, acceptable, and generally well received by mothers or caregivers and children aged 6 to 59 months in Mexico. However, strategies are required to address consumption barriers, including strengthening IYCF counselling, community and family awareness about IAM, and reinforcing healthcare-based local support mechanisms at the primary care level during treatment, as well as adapting operational approaches to Mexico’s sociocultural and territorial diversity.

Local development and production of RUTF should be strongly encouraged, as its effectiveness and high acceptability have been demonstrated in other contexts [39]. Expanding product availability could support national scale-up and inform public policy decision-making. Evidence-based recommendations for RUTF program implementation include:

- Implement cultural adaptation strategies that address regional taste preferences and dietary practices, particularly in populations unfamiliar with peanut-based products.
- Strengthen community-based follow-up and referral systems, including home visits and active case-finding, to prevent loss to follow-up.
- Train health and community personnel on the appropriate use of RUTF and culturally relevant communication strategies to promote adherence.
- Integrate acceptability and adherence indicators into routine health and nutrition monitoring systems as part of national child malnutrition guidelines.

Close monitoring and intensive counselling of health providers are essential for the success of MAM and SAM treatment at home. Therefore, government programs should consider incorporating social and behavioural change communication (SBCC) to motivate the health personnel and parents and ensure optimal compliance [40].

## Figures and Tables

**Table 1:** Sociodemographic and anthropometric characteristics of the sample and nutritional diagnosis of the participating children. Mexico, 2024.

Mother/Primary Caregiver (PC)	Chihuahua (n = 13)	Guerrero (n = 31)	Chiapas (n = 22)	Total (n = 66)
Age of PC (Mean ± SD)	28.3 ± 5.3	29.4 ± 6.7	30.3 ± 6.8	29.5 ± 6.4
Marital Status (%) ^a^				
Single	3 (23.0)	1 (3.2)	—	4 (6.0)
Married	6 (46.1)	17 (54.8)	8 (36.3)	31 (46.9)
Cohabiting	4 (30.7)	13 (41.9)	14 (63.6)	31 (46.9)
Education Level (%)				
Basic or less	9 (69.2)	12 (40.0)	13 (59.0)	34 (52.3)
Middle/high school	4 (30.7)	14 (46.6)	9 (40.9)	27 (41.5)
University or higher	—	4 (13.3)	—	4 (6.1)
Occupation of PC (%)				
Employed/with income	1 (7.6)	1 (3.23)	—	2 (3.0)
Housewife	10 (76.9)	25 (80.6)	21 (95.4)	56 (84.8)
Farmer	2 (15.3)	1 (3.2)	1 (4.5)	4 (6.0)
Merchant	—	3 (9.6)	—	3 (4.5)
Other	—	1 (3.2)	—	1 (1.5)
Indigenous Language (%)				
Yes	7 (53.8)	10 (32.3)	12 (54.5)	29 (43.9)
No	6 (46.1)	21 (67.7)	10 (45.4)	37 (56.0)
Socioeconomic Level *^a^				
A/B (High)	—	—	—	—
C (Middle)	2 (15.3)	16 (53.3)	4 (18.1)	22 (33.8)
D/E (Low/Very low)	11 (84.6)	14 (46.6)	18 (81.8)	43 (66.1)
**Child Participant**	**Chihuahua (n = 13)**	Guerrero (n = 30) **	**Chiapas (n = 22)**	**Total (n = 65)**
Age (months, Mean ± SD) ^a^	17.1 ± 16.0	21.2 ± 13.6	30.4 ± 15.6	23.5 ± 15.4
6–24 months (%)	11 (84.6)	21 (70.0)	9 (40.9)	41 (63.0)
24–59 months (%)	2 (15.3)	9 (30.0)	13 (59.0)	24 (36.9)
Sex (%)				
Boys	8 (61.5)	16 (51.6)	11 (50.0)	35 (53.0)
Girls	5 (38.4)	15 (48.3)	11 (50.0)	31 (46.9)
Weight (kg, Mean ± SD) ^a^	7.4 ± 2.3	8.0 ± 2.0	9.2 ± 1.9	8.3 ± 2.1
Height/Length (cm ± SD) ^a^	71.4 ± 12.9	76.9 ± 9.9	82.8 ± 11.2	77.8 ± 11.6
Mid-Upper Arm Circumference	12.4 ± 1.2	12.2 ± 0.3	12.2 ± 0.2	12.2 ± 0.5
Nutritional Status Diagnosis ^a^				
Severe Acute Malnutrition (SAM)	4 (30.7)	—	1 (4.5)	5 (7.6)
Moderate Acute Malnutrition (MAM)	9 (69.2)	30 (100.0)	21 (95.4)	60 (92.3)

^a^ Valor *p* < 0.05 using ANOVA for continuous variables and chi-2 for categorical variables. * Socioeconomic level: A/B: High; C: Middle; D/E: Low/very low. ** One record from Guerrero was excluded from the analysis because the child was born preterm (<37 weeks of gestation) and therefore did not meet the study’s inclusion criteria.

**Table 2:** Estimated mean results of the hedonic scale applied to mothers/primary caregivers regarding their own perception and their children about the consumption of the RUTF treatment. Mexico, 2024.

		Hedonic Scale Category (%) **				
RUTF Organoleptic Characteristic		Very Unpleasant	Unpleasant	Neutral	Pleasant	Very Pleasant
Texture	Mothers/primary caregivers	0.6	3.3	14.8	38.7	42.5
	Children	1.9	7.1	7.1	42.9	40.8
Overall likability	Mothers/primary caregivers	0.0	1.3	6.3	32.4	59.9
	Children	1.9	4.6	1.9	39.5	52.0
Appearance	Mothers/primary caregivers	0.0	3.2	19.6	29.0	48.2
	Children	0.6	5.8	9.6	39.6	44.3
Smell	Mothers/primary caregivers	0.6	6.5	8.3	35.1	49.4
	Children	0.6	8.5	9.6	42.9	38.2
Taste	Mothers/primary caregivers	0.6	3.3	9.6	40.3	46.1
	Children	2.5	7.2	1.9	33.8	41.2

*p*-value > 0.05. ** Hedonic scale results include data collected from baseline to follow-up 3. The fourth follow-up was conducted only in one state (n = 10) and was therefore excluded from this comparative analysis to ensure consistency across study sites.

## Data Availability

The data presented in this study are available on request from the corresponding author A.B.A. due to privacy and legal issues of safeguarding participants’ information.

## Supplementary Materials

The following supporting information can be downloaded at: https://www.mdpi.com/article/10.3390/nu18030444/s1, File S1: Information on RUTF consumption.

## Abbreviations

The following abbreviations are used in this manuscript:

RUTFs	Ready-to-use therapeutic foods
MAM	Moderate acute malnutrition
SAM	Severe acute malnutrition
IAM	Infant acute malnutrition
WHZ	Weight-for-height z-scores
WLZ	Weight-for-length z-scores
SD	Standard deviations
ENSANUT	National Health and Nutrition Survey
WHO	World Health Organization
MUAC	Mid-Upper Arm Circumference
INSP	National Institute of Public Health
TFA	Theoretical Framework of Acceptability
SES	Socioeconomic status
AMAI (in Spanish)	Mexican Association of Market Research Agencies
HAZ	Height-for-age z-scores
LAZ	Length-for-age z-scores
SQ-LNS	Small-quantity lipid-based nutrient supplements
IYCF	Infant young children feeding
SBCC	Social and behavioural change communication

## Appendix A

### Appendix A.1

**Table A1:** Mean weight, height/length, and MUAC by follow-up and number of MAM or SAM cases treated with RUTF. Mexico, 2024.

	n	Weight SD (kg)	Height/Length SD (cm)	MUAC SD (cm)
Baseline	65	8.33	77.85	12.27
Follow-up 1	51	8.81	78.89	12.94
Follow-up 2	45	9.40	78.55	13.31
Follow-up 3	23	8.72	77.96	13.27
Follow-up 4	10	8.67	77.80	13.15

### Appendix A.2

**Table A2:** Barriers and facilitators for acceptability and adherence to RUTF treatment in cases of MAM or SAM. Mexico, 2024 *.

RUTF Treatment	Interviewer	Example Quotes
Frequency and administration	“I: How many days a week did you give it to him/her?”	“PC: Every day because those were the instructions.” PC_Chiapas
	“I: What was the strategy for your child to be able to eat the therapeutic food?”	“PC: Well, the first few days I would take out the spoon with treatment from another bowl with food that he/she liked so that he/she could see that it wasn’t the treatment but the other food. (…) He/She had two small bowls. In one bowl, he/she had the food he/she liked and in the spoon the treatment. In the other small bowl, I would grab a little bit of that, and then I would give him/her the other one.” PC_Chiapas
	“I: How many times a day did you separate it?”	“PC: About four or five times.” PC_Chiapas
	“I: On a normal day, what amount of sachets or how many sachets did your child consume?”	“PC: At first, he/she would almost finish it, but later, there were times when a little less than half, or maybe I think half.” PC_Chiapas
Barriers	“I: Did he/she have any discomfort or anything?”	“PC: Yes, he/she had stomach discomfort, but from then on, as his/her stomach got used to it and reacted well, until now I haven’t given him/her any medication or anything.” CP_Guerrero
	“I: Did you notice if, in those days when you started giving the therapeutic food, he/she had any reaction?”	“PC: It made him/her vomit, and then he/she would say that his/her belly hurt, so I think that’s why I decided not to give it to him/her.” PC_Chihuahua
Facilitators	I: You mentioned that your child had diarrhea for a few days. Do you associate it with the RUTF? Or do you think it was caused by something else? What do you think it was?	“PC: No, I think it was because I left my child with my sister, and she gave him tap water, and I believe that’s why he had that (diarrhea).” PC_Chihuahua
	“I: And what did you like about the treatment?”	“PC: I liked that, above all, it’s easy to give to him/her. It doesn’t have another flavor so bitter or so strong like medication; it’s something easy that the child can eat.” PC_Guerrero
	“I: Did you notice if he/she accepted it easily or struggled to eat it?”	“PC: At first, since it was a new product for him/her, yes, at first yes, the first week, almost like three, four days we struggled to give it to him/her little by little, there were more spoonfuls, but later, he/she himself/herself, we would take it out of the corner, and he/she alone would start to eat it.” PC_Guerrero
	“I: And how do you feel about those results?”	“PC: Happy and content because I see my baby; there he/she goes little by little.” PC_Guerrero
	“I: If you had the opportunity to continue giving the treatment to your babies for longer, would you like it?”	“PC: Yes. So that they are well, so that they are healthy.” PC_Guerrero

* Legend: I = Interviewer; PC = Primary caregiver.
