# Supplementary material for: Acceptability and Adherence to Ready-to-Use Therapeutic Foods (RUTFs) Treatment in Cases of Moderate and Severe Acute Malnutrition in Children from Rural and Indigenous Communities in Mexico
Source: Nutrients. 2026 Jan 29;18(3):444. doi: 10.3390/nu18030444 (PMC12899508; doi:10.3390/nu18030444)
Supplement: Supplementary file 1 [file nutrients-18-00444-s001.zip › nutrients-4074760-supplementary.pdf]

ADDITIONAL NOTES

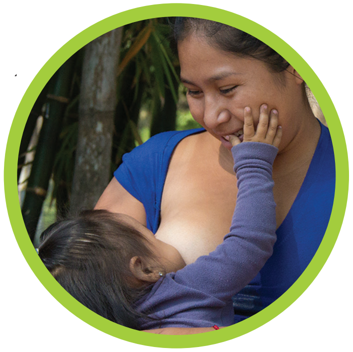

- RUTF does not replace breastfeeding or the child's regular daily meals.
- RUTF should be offered in small amounts several times throughout the day.
- It is a medical treatment and must not be shared with other family or household members.
- It contains peanuts, milk, dairy products, and soy, so children with allergies to these foods must not consume it.
- The supplement should not be mixed with other foods or liquids.
- Maintain an attentive and caring attitude while feeding the child; it is recommended to talk, sing, and play to stimulate appetite and development.

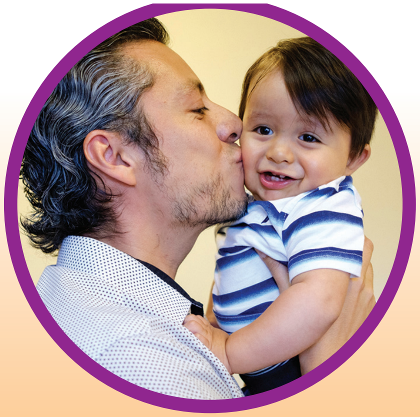

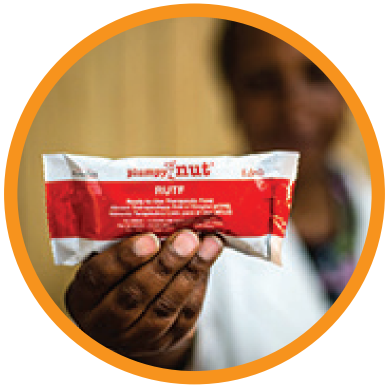

RUTF can be eaten directly from the sachet by children on their own, without help from a mother, father, or caregiver.

- Once opened, the sachet can be consumed within 24 hours. It must be stored in a closed container and kept in a cool, dry place protected from sunlight, below 30°C (86°F).
- If the child has diarrhea, do not stop offering RUTF. These conditions may appear at the beginning of treatment but will disappear. It is important to continue giving RUTF.
- Let the child drink as much water as needed while eating RUTF.

References

1. WHO. WHO guideline on the prevention and management of wasting and nutritional oedema (acute malnutrition) in infants and children under 5 years. 2023.

2. Schoonees A, et al. Ready-to-Use Therapeutic Food for home treatment of severe acute malnutrition in children 6–59 months. Cochrane Database Syst Rev. 2019; CD009000.

Instituto Nacional de Salud Pública

unicef para cada infancia

The development of these materials was made possible thanks to the technical and financial contribution of UNICEF.

Use of Ready-to-Use Therapeutic Food (RUTF)

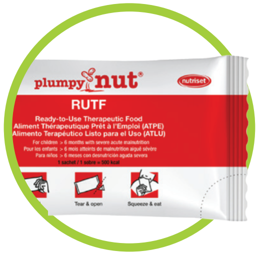

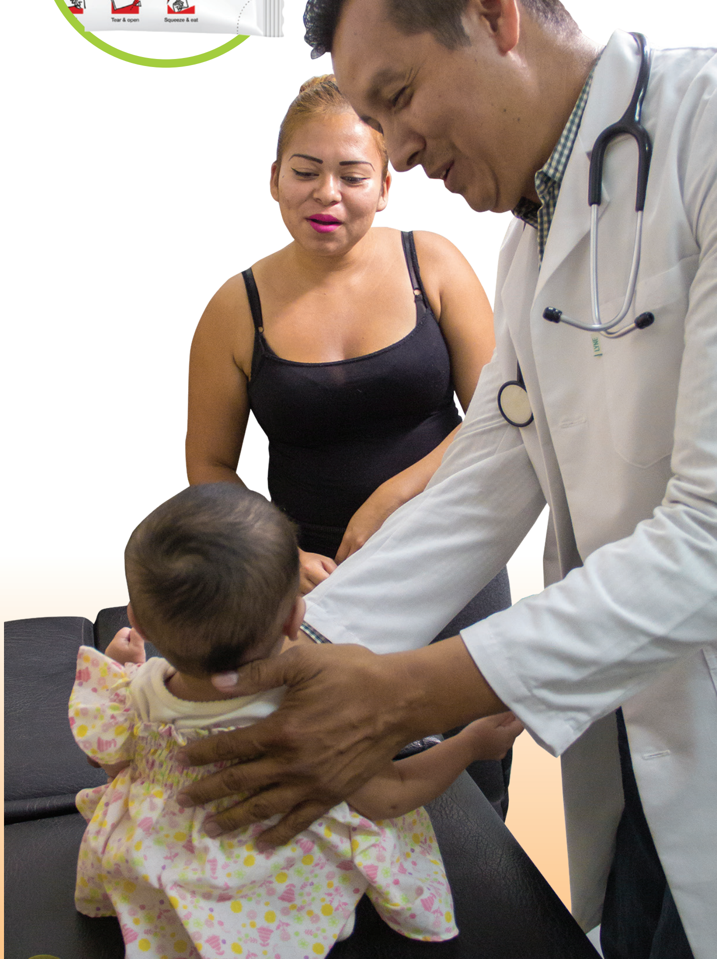

What is it?

RUTF is a medical treatment made from peanut paste, powdered milk, vegetable oil, sugar, vitamins, and minerals.

Who should consume it?

It is recommended for children aged 6 to 59 months (6 months up to 5 years of age) diagnosed with moderate or severe acute malnutrition.

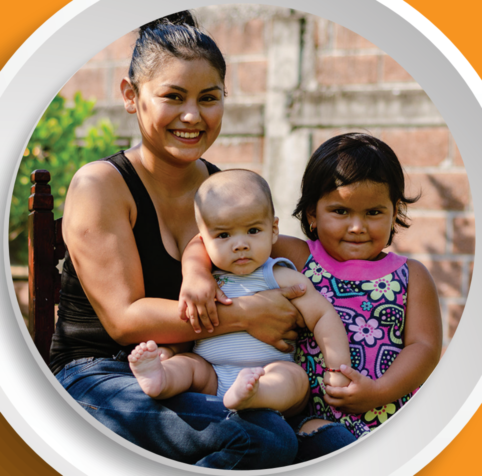

WHY IS IT IMPORTANT TO TREAT MALNUTRITION?

A.
It affects the child's health, growth, and development.

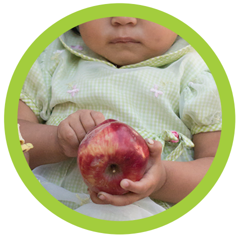

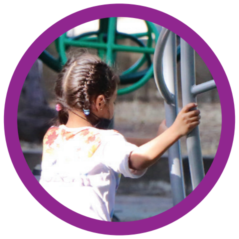

B.
Children are at greater risk of illness and death from preventable causes.

C.
If not treated in time, it can affect the child's physical and mental development.

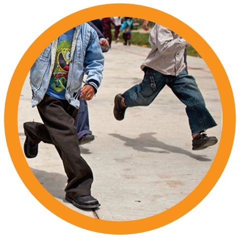

D.
It may affect school performance in children.

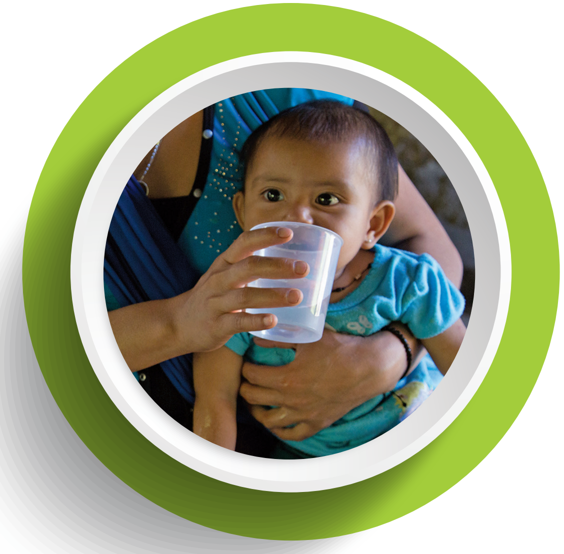

Instructions for Consumption or Use of RUTF

1. Wash the child's face and hands with soap and water.

2. Wash or clean the RUTF sachet.

3. Cut one corner of the sachet.

4. Let the child eat the RUTF by squeezing the sachet. This must be done under the supervision of the mother, father, or primary caregiver, especially in children under 1 year of age. A small spoon may also be used to offer RUTF.

5. Always offer the child plenty of clean, safe drinking water while consuming RUTF.
